# An Optimised Protocol Harnessing Laser Capture Microdissection for Transcriptomic Analysis on Matched Primary and Metastatic Colorectal Tumours

**DOI:** 10.1038/s41598-019-55146-2

**Published:** 2020-01-20

**Authors:** Chin-Ann J. Ong, Qiu Xuan Tan, Hui Jun Lim, Nicholas B. Shannon, Weng Khong Lim, Josephine Hendrikson, Wai Har Ng, Joey W. S. Tan, Kelvin K. N. Koh, Seettha D. Wasudevan, Cedric C. Y. Ng, Vikneswari Rajasegaran, Tony Kiat Hon Lim, Choon Kiat Ong, Oi Lian Kon, Bin Tean Teh, Grace H. C. Tan, Claramae Shulyn Chia, Khee Chee Soo, Melissa C. C. Teo

**Affiliations:** 1 Department of Sarcoma, Peritoneal and Rare Tumours (SPRinT), Division of Surgery and Surgical Oncology, National Cancer Centre Singapore, 11 Hospital Drive, Singapore, S169610 Singapore; 2 Cancer and Stem Cell Biology, Duke-NUS Medical School, 8 College Road, Singapore, 169857 Singapore; 3 Division of Medical Sciences, National Cancer Centre Singapore, 11 Hospital Drive, Singapore, S169610 Singapore; 4 Department of Anatomical Pathology, Singapore General Hospital, 20 College Road, Singapore, S169856 Singapore

**Keywords:** Tumour biomarkers, Transcriptomics

## Abstract

Generation of large amounts of genomic data is now feasible and cost-effective with improvements in next generation sequencing (NGS) technology. Ribonucleic acid sequencing (RNA-Seq) is becoming the preferred method for comprehensively characterising global transcriptome activity. Unique to cytoreductive surgery (CRS), multiple spatially discrete tumour specimens could be systematically harvested for genomic analysis. To facilitate such downstream analyses, laser capture microdissection (LCM) could be utilized to obtain pure cell populations. The aim of this protocol study was to develop a methodology to obtain high-quality expression data from matched primary tumours and metastases by utilizing LCM to isolate pure cellular populations. We demonstrate an optimized LCM protocol which reproducibly delivered intact RNA used for RNA sequencing and quantitative polymerase chain reaction (qPCR). After pathologic annotation of normal epithelial, tumour and stromal components, LCM coupled with cDNA library generation provided for successful RNA sequencing. To illustrate our framework’s potential to identify targets that would otherwise be missed with conventional bulk tumour sequencing, we performed qPCR and immunohistochemical technical validation to show that the genes identified were truly expressed only in certain sub-components. This study suggests that the combination of matched tissue specimens with tissue microdissection and NGS provides a viable platform to unmask hidden biomarkers and provides insight into tumour biology at a higher resolution.

## Introduction

High-throughput next-generation sequencing (NGS) is a powerful strategy to study cancer progression at the molecular level, where ribonucleic acid sequencing (RNA-Seq) is becoming the preferred method for comprehensively characterising global transcriptome activity^1^. By comparing the transcriptomes, differential expression of genes in discrete cell populations can be easily identified. This approach has emerged as a useful tool for characterising the transcriptomes and molecular signatures of tissues-of-interest via RNA or protein profiling, which may reflect their functionality^2^. In recent years, large genomic consortiums such as The Cancer Genome Atlas (TCGA) and The International Cancer Genome Consortium (ICGC) have been established^1^. With these publically available large-scale integrated data, much effort have been placed into identifying putative prognostic biomarkers using NGS, however, few have made it to clinical practice^3^. A plausible reason for this could be due to the limitations of conventional sequencing methods which analyse whole tumour biopsies^4^. Conventional sampling of a cancerous mass often results in a collection of varied cell types as tumour cells are frequently intermixed with stromal, inflammatory and epithelial cells. This leads to great difficulty in obtaining pure samples of malignant cells, and as a result complicate the genetic evaluation of these cells^5,6^. To gain deeper understanding of biological processes and disease mechanisms, it is vital to elucidate gene expression of discrete cellular populations found in whole tumour samples^7,8^.

Laser capture microdissection (LCM) is a unique method which allows the segregation of pure cell populations from defined anatomical locations instead of whole tumour biopsies^9^. Using microscopy to identify specific compartments of tissue, laser is used to precisely dissect the samples-of-interest, following which nucleic acids can be extracted from these pure cellular populations and used in downstream experiments, revolutionizing molecular analyses of intricate tissue samples^10–13^. In conjunction with RNA extraction methods, LCM has been used to ensure effective processing of samples, and analyse transcriptomes of discrete tumour cell populations and low-quantity cellular populations in tissues^14^. In comparison to other techniques for isolation of cells, LCM allows for the retention of positional information of cells without requiring tissue dissociation. Additionally, there is no need for live cells with genetic labels^15^.

Although LCM is widely utilized on a variety of tissue types, obtaining high-quality RNA remains difficult and necessitates for strict protocols which are critical for downstream analyses^16,17^. Furthermore, tissue handling time plays a critical role in the preservation of RNA integrity. In a selected group of patients who undergo cytoreductive surgeries (CRS) that usually take numerous hours, it is particularly challenging to harvest specimens with optimal RNA integrity and would require a streamlined workflow to ensure that specimens are processed promptly after being surgically removed. Hence, a feasible approach using targeted LCM that precisely captures areas of interest to achieve accurate RNA sequencing with minimal starting material is pertinent in light of the challenges of procuring adequate amounts of tissue samples.

In this protocol paper, we aim to demonstrate a validated workflow incorporating LCM for robust transcriptomics analysis in the era of NGS compared to the conventional bulk tumour sequencing. There is currently no literature on the use of LCM to reproducibly isolate high-quality RNA from systematically collected matched tumours and its downstream applications specifically in colorectal and Krukenberg tumours. Furthermore, there is a lack of publicly available data, such as on TCGA, which are matched. As such, differences in gene expression in unmatched normal and tumour samples may not be truly representative of upregulated genes or signaling pathways in malignant cells. We have established a methodology developed for RNA-Seq that couples LCM with Nugen Ovation FFPE RNA Multiplex System for efficient sequencing of RNA in microdissected clinical samples from colorectal and Krukenberg tumours which have not been demonstrated before. The optimized workflow for RNA isolation and sequencing from LCM samples of matched normal mucosal, colorectal and Krukenberg tumours was determined. qPCR verification on several chosen targets was then performed to evaluate the quality of RNA-Seq data. Further confirmation of the spatial expression by immunohistochemistry (IHC) was performed to validate the target *in situ* and to assess the accuracy of the LCM. Finally, to determine if LCM samples performed better compared to whole tumour samples, qPCR analyses on both microdissected and non-microdissected matched trios of normal-primary-metastatic samples were conducted.

## Results

### Optimisation of pipeline

**Figure 1:**
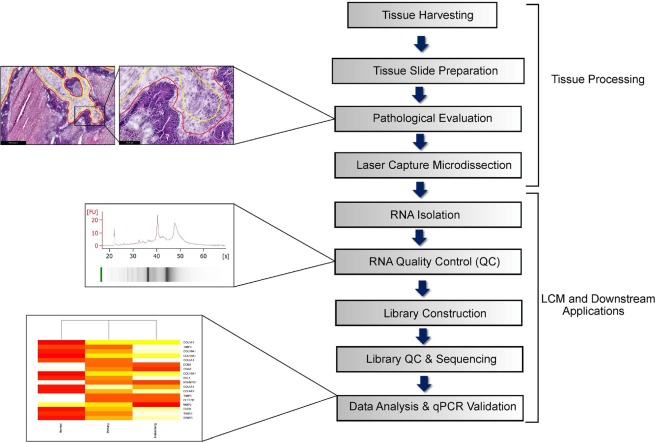
Schematic illustration of the workflow.

The workflow consisting of major steps such as LCM, RNA isolation and RNA sequencing is depicted in Fig. 1. Each process has been optimized to ensure seamless flow of tissue collection and tissue processing to preserve and obtain good quality RNA for downstream applications like RNA sequencing and qPCR validation. Since RNA starts degrading once the tissue is removed from the body, tissue handling time was kept to minimum throughout the procedure. We ensured that the time of tissue resection to tissue harvest was carried out as quickly as possible and the tissue was snap frozen immediately before bringing the sample to our laboratory for further processing. Additionally, the time from staining the sample to completion of LCM was strictly maintained to less than 30 mins for each slide. The temperature for tissue storage is also a critical component for maintaining high RIN. For long-term storage, our samples were kept in a −80 °C freezer. Alternatively, we stored the samples in liquid nitrogen or dry ice for short-term storage. The usage of wet ice for temporary storage was not suitable. To minimize freeze-thaw cycles of our eluted RNA, which would ultimately degrade them, we stored eluted RNA in aliquotes. Lastly, we applied RNase Away to ensure a clean working area and used nuclease/RNase free water for all our experiments to prevent contamination.

### Patient samples and LCM

**Figure 2:**
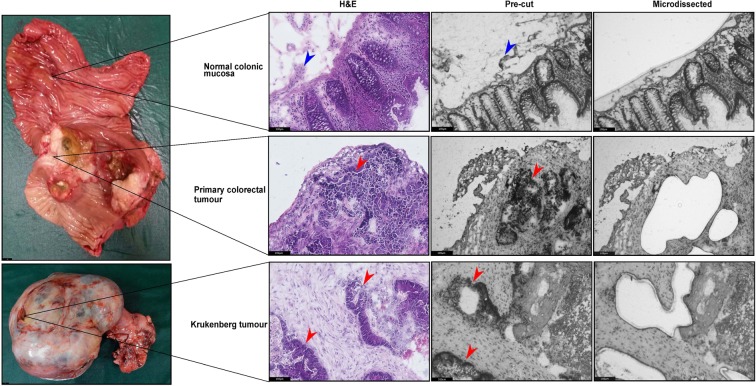
Representative images of surgical samples harvested in trio (normal-primary-metastasis), H&E stained reference section (10X) and LCM sections (10X). Red arrows represent tumour epithelial cells while blue arrows represent the stromal cells.

Multisampling of colorectal and Krukenberg tumour samples in conjunction with the associated normal mucosa was performed (Fig. 2). Normal colonic mucosa was collected to serve as control in the gene expression analysis. Analyzing these samples in trio - normal mucosa, primary tumour and metastasis - could provide insights to the biological process that may be masked by tumour centric analysis. We identified four patients who underwent cytoreductive surgery for resection of Krukenberg tumours. Among these patients, complete normal-primary-metastasis sample trio were obtained for Patients 1 and 2. Matched primary colon tumour and Krukenberg tumour specimens were collected from Patient 3 while only Krukenberg tumour was available for Patient 4 (Supplementary Table S1). Biopsies were systematically harvested and snap frozen in liquid nitrogen immediately upon resection to preserve good morphology and RNA integrity of the specimen for histological assessment and transcriptomic analysis respectively. The RNA quality will affect the success of the downstream processes, highlighting the importance of proper tissue handling. The tissues-of-interest were marked by the clinical pathologist on digitalized haematoxylin and eosin (H&E) stained slides (Supplementary Fig. S1). During microdissection, the cresyl violet-eosin quick staining protocol provided good morphological resolution of the tissue samples. Using the pathologically annotated image as reference, areas-of-interest were identified and microdissected (Fig. 2).

### RNA integrity number (RIN) analysis and RNA quantification

**Figure 3:**
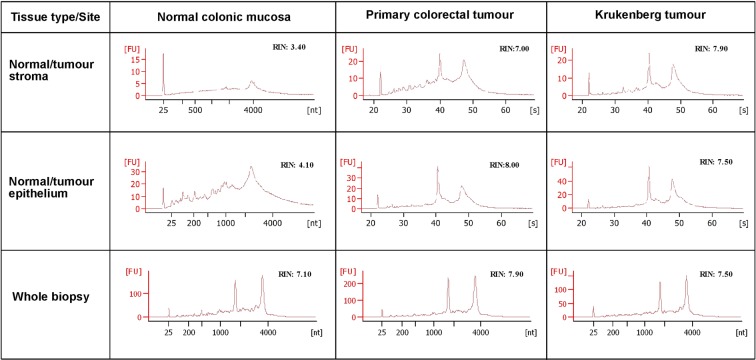
Total RNA capillary electrophoresis Bioanalyzer electrochromatograms from LCM samples from patient 1. These diagrams show the differences in quality of RNA obtained from different LCM samples. *FU, fluorescence units; RIN, RNA integrity number; nt, nucleotide; s, seconds*.

Total RNA was extracted from the microdissected tissues. The quality and degree of degradation of extracted RNA was appraised via RNA integrity number (RIN) before amplification. The quality of RNA integrity is determined by an algorithm applied to electrophoretic RNA analysis and is expressed as the RIN that ranges between 1 (totally degraded) to 10 (intact). The automated electrophoresis Bioanalyzer system provided the RIN for the 20 samples, with a mean value of 5.53 ± 1.73 (s.d.), indicating relatively good RNA quality (Fig. 3 and Table 1).

**Table 1:** RNA integrity and quantity of microdissected samples.

SN	Sample ID	RIN	Total RNA input 5 µl (ng)
1	Pat 1 Colonic Mucosa Stroma	7.9	4.82
2	Pat 1 Colonic Mucosa Epithelial	5	59.22
3	Pat 1 Primary CRC Stroma	7	47.20
4	Pat 1 Primary CRC Tumour	8	41.60
5	Pat 1 Krukenberg Stroma	7.9	28.85
6	Pat 1 Krukenberg Tumour	7.5	57.00
7	Pat 2 Colonic Mucosa Stroma	5	3.80
8	Pat 2 Colonic Mucosa Epithelial	3.1	16.17
9	Pat 2 Primary CRC Stroma	5.2	11.97
10	Pat 2 Primary CRC Tumour	6.3	24.08
11	Pat 2 Right Krukenberg Stroma	4.5	12.55
12	Pat 2 Right Krukenberg Tumour	7.1	28.96
13	Pat 2 Left Krukenberg Stroma	5.9	10.30
14	Pat 2 Left Krukenberg Tumour	7.1	20.76
15	Pat 3 Primary CRC Stroma	3	20.05
16	Pat 3 Primary CRC Tumour	4.2	10.06
17	Pat 3 Krukenberg Stroma	5	56.04
18	Pat 3 Krukenberg Tumour	4.1	81.14
19	Pat 4 Krukenberg Stroma	3	17.08
20	Pat 4 Krukenberg Tumour	3.7	23.87
	**Average**	5.5	28.8
	**S.D**.	1.7	21.3

For all the samples, the overall mean value of the RNA quantity in 5 µl of eluent was 28.77 ng ± 21.31 (s.d.), whereby all except two samples (normal mucosa stroma) were well above the recommended input quantity by the Manufacturers’ guidelines (Fig. 3 and Table 1). This threshold was overruled for the sample below the lower limit of 10 ng and was used to construct a library along with all the other samples.

### Sequencing data quality control

**Figure 4:**
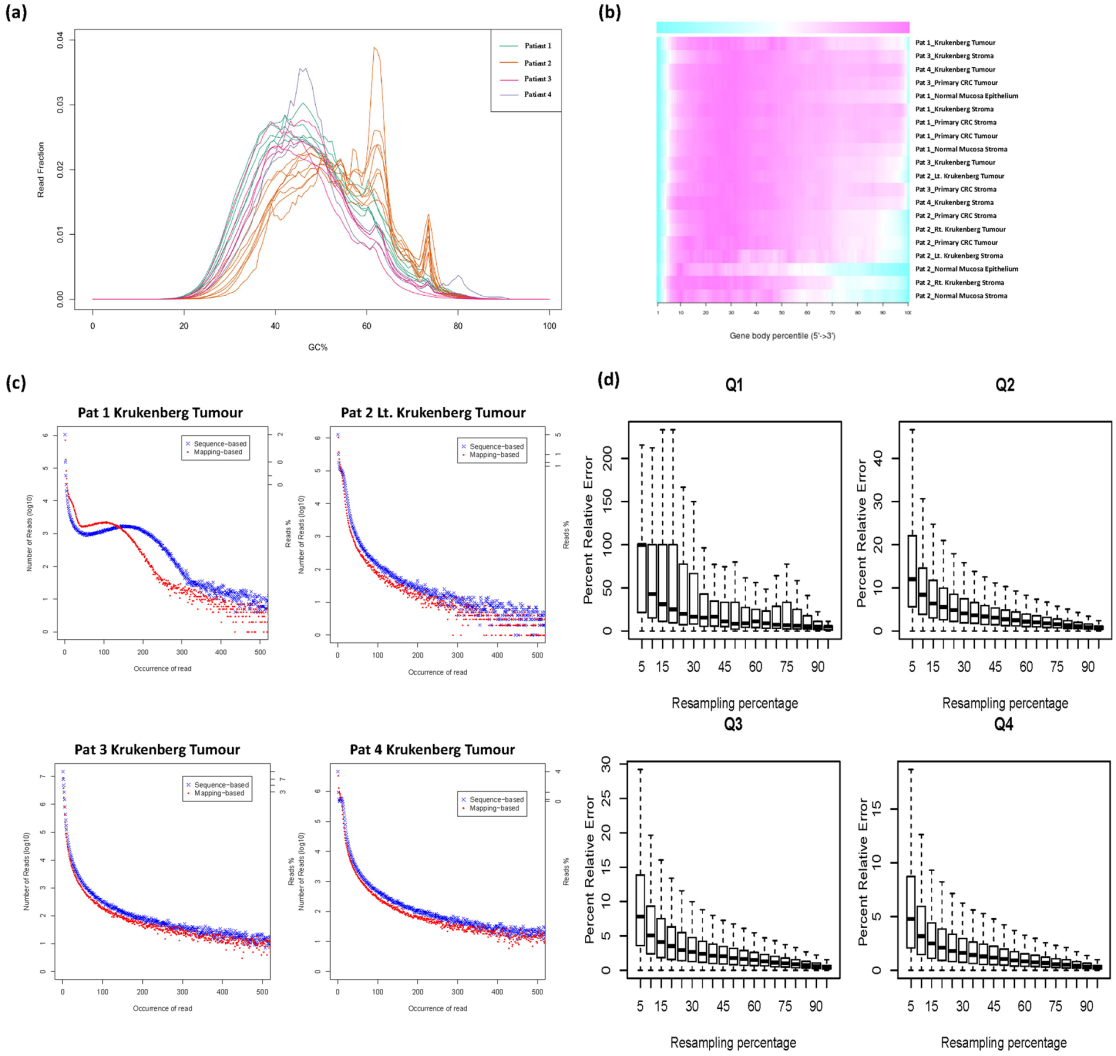
(**a**) GC Content of LCM samples. There is an expected normal distribution of % GC content across sequencing reads in all 20 samples. (**b**) Heat map of the gene body coverage of all libraries. Overall, the 5′ end has higher coverage compared to the 3′ end. This 5′ to 3′ bias is consistent between all samples indicating comparable expression values amongst them. The graph was generated using the aligned reads for each library and inputted into the geneBodyCoverage.py script from the RseQC package. (**c**) Representative plots illustrating the distribution of duplicated reads relative to the total number of sequences for all libraries. (**d**) Saturation plot of the colorectal tumour component, illustrating percent relative error versus resampling fraction for transcripts grouped by expression quartile (<25^th^ percentile, between 25^th^ and 50^th^ percentile, between 50^th^ and 75^th^ percentile, and above 75^th^ percentile).

Raw RNA-Seq data was aligned to the human reference transcriptome (hg19) and the quality of the sequencing data assessed using RSeQC. We first studied the distribution of GC content across all reads in each sample. Most samples had the expected normal distribution of GC content, with some samples from patient 2 having additional peaks, possibly due to contamination of sequencing adapters or other factors related to sample preparation (Fig. 4a). In addition, we examined the presence of 5′to 3′ bias in the data by applying a gene body coverage analysis on the mapped reads for all libraries using the geneBodyCoverage.py script in RSeQC. No significant 5′or 3′ end bias was reported based on the gene body coverage analysis (Fig. 4b). We note again that samples from patient 2 had reduced coverage at the 3′ end. The duplication levels relative to the total number of sequences show a distribution that is skewed to the right of the graph, indicating that most of the libraries demonstrate good library diversity. However, some of the samples exhibit higher levels of duplication which may be due to the contamination of the library possibly from residual ribosomal RNA as observed from the report of overrepresented sequences (Fig. 4c and Supplementary Fig. S2a). We then performed a saturation analysis to check if the sequencing coverage is sufficient to give a good representation of the intrinsic transcriptomic profile (Fig. 4d and Supplementary Fig. S2b). The saturation plots indicate that the number of reads is sufficient to accurately quantify even low-abundance transcripts.

### Analysis of the RNA-Seq results

**Figure 5:**
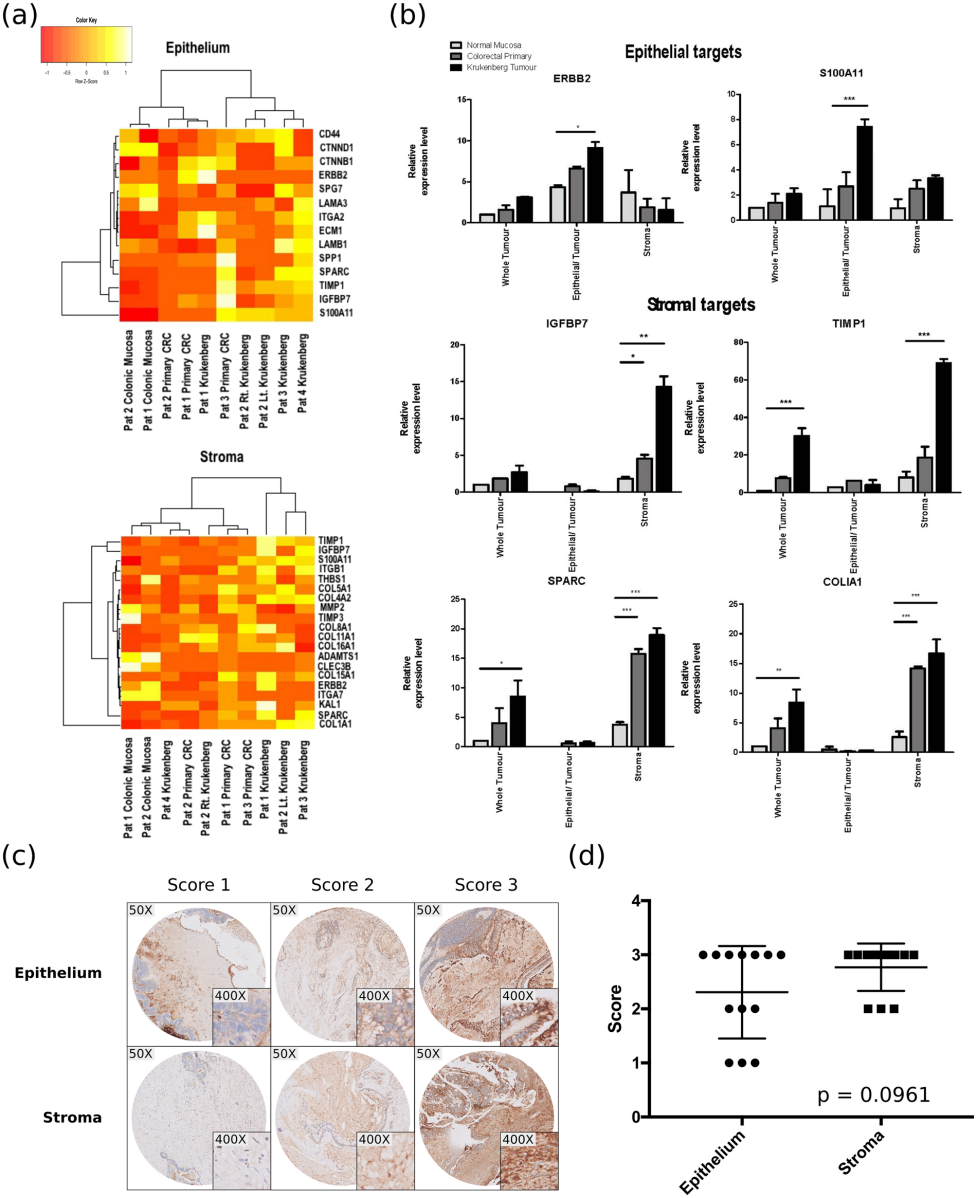
Validation of RNA-Seq data by qPCR analysis of LCM samples. (**a**) Heat map illustrating the epithelial and stromal transcriptomic landscape of all the RNA-Seq samples across patients. Each row is normalized using Z-score. (**b**) Results illustrating the expression patterns of epithelial targets (ERBB2 and S100A11) as well as stromal targets (IGFBP7, TIMP1, SPARC & COLIA1) across samples and tissue types. These targets were upregulated specifically in the respective compartments. The data was normalised to *β*-Actin and analysed using one-way ANOVA. Results are the mean of three biological replicates and standard deviations are shown. (**c**) Representative immunohistochemical (IHC) staining of IGFBP7, illustrating the scores 1 to 3 for epithelial and stromal staining intensity scoring in our Krukenberg samples. (**d**) Distribution of intensity scoring for epithelial and stromal staining in Krukenberg samples (*p* = 0.0961). *p < 0.05, **p < 0.01, ***p < *0.001*.

To compare the transcriptomic landscape of the samples, we applied unsupervised clustering analysis to the expression data generated from RNA-Seq. Figure 5a demonstrates the variability of transcriptomic signatures of the various samples both in the epithelial and stromal components. Briefly, expression data of both the stroma and epithelium revealed that the samples from the 4 patients formed groups corresponding to normal, primary tumour and Krukenberg with a couple of exceptions in the samples. This observed pattern suggests that there is a common molecular modification process driving the formation of cancers as the normal cells progress to form tumours and eventually gain the ability to metastasize. The modification of the transcriptome does not merely occur in the epithelial cells. It also happens in the stroma.

Using the RNA sequencing data of the epithelial component of the tumours and normal mucosa, the 2D principal component analysis (PCA) plots revealed that the principal components (PC) 1 and 2 separated tumour versus non-tumour epithelium into two groups. This observation is further supported by the distance matrix of the epithelium that demonstrated two distinct clustering that corresponds to tumour and non-tumour samples in general (Supplementary Fig. S3). Based on these findings, it is clear that the normal colonic mucosa exhibits a completely different transcriptomic profile compared to both the primary tumours and Krukenberg tumours (Supplementary Fig. S3). Specifically, in patient 1, the transcriptomic profile of the Krukenberg tumours differs greatly from that to the primary tumour but clustered with the Krukenberg tumours from the 3 other patients. In patients 2 and 3, the transcriptomic profile of the Krukenberg tumours is more similar to the primary tumour compared to patient 1, suggesting potential differences in the biological aggressiveness across patients with colorectal tumours that metastasize to the ovary (Krukenberg).

On a similar note, as suggested by the PCA plot and distance matrix in Supplementary Fig. S3, the stroma of the normal colonic mucosa can be separated from that of the tumour stroma using unsupervised clustering of the respective transcriptomic profiles. This observation reinforces the fact that the transcriptomic profile of the tumour microenvironment is different from the stroma of the normal colonic mucosa. In general, the PCA plot revealed that the stromal transcriptome of the primary tumours across the 3 patients as well as the Krukenberg tumours of patients 1 and 4 are similar when taking into consideration the PC1, suggesting a common molecular behavior across these metastatic tumours. In patients 2 and 3, the transcriptomic profiles of stroma derived from the Krukenberg tumours are dissimilar to that of the stroma obtained from the respective primary tumours. These biological differences may explain differences in the priming of the tumour microenvironment to facilitate metastatic spread in different patients.

### qPCR verification of RNA-Seq results

To illustrate proof-of-principle that LCM is a means to unveiling hidden biomarkers which could not be picked up by conventional whole biopsy analysis, we performed technical verification of the RNA-Seq expression data via qPCR on samples from a single patient, using selected targets that were deemed biologically relevant in the literature, and showed different expression patterns. For instance, IGFBP7 is a known stromal marker in many epithelial cancers, and elevated expression is associated with disease progression and poorer overall survival^18–20^. COL1A1, a fibrillar collagen encoding gene found in the stroma of multiple cancers, plays a role in tumour proliferation, invasion, migration and angiogenesis^18,21–23^. Upregulation of ERBB2 (HER2) is associated with aggressive tumours, and poorer overall and disease-free survivals in breast cancer^24,25^. We subjected these differentially expressed genes, namely ERBB2, SPINK1, CEACAM1, SPARC, TIMP1, IGFBP7, COL1A1, ENO1, VEGFA, S100A11 and PLA2G2A, to validation by qPCR using specific primers (Supplementary Table S3). qPCR relative expression data of these selected targets corresponded with the expression patterns identified by the RNA-Seq data of microdissected samples (Fig. 5b and Supplementary Fig. S4). Apart from confirming targets, such as ERBB2 and S100A11, which are exclusively upregulated in the tumour epithelial cells, qPCR data confirmed that stromal targets such as SPARC, TIMP1, IGFBP7, COL1A1 were upregulated specifically in stromal components and not in epithelial components (Fig. 5b).

To investigate the impact of microdissection in target calling, we performed qPCR on microdissected samples as well as matched whole tumour biopsies which were not subjected to microdissection. Comparing gene expression data between microdissected samples and whole biopsies, merely 1 out of 9 genes had matched gene expression patterns across the two groups of comparison, whereby TIMP1 was unanimously identified as a metastasis specific gene (*p* ≤ 0.001) in both analyses. Interestingly, our results demonstrated that whole tumour biopsies may not provide enough resolution to identify important targets that might be hidden in sub-compartments of tissues. For example, expression of IGFBP7 was insignificant in whole tumour analysis while homogenous stromal analysis showed significantly higher expression of IGFBP7 in both primary tumour and metastasis with 2.719 (*p* ≤ 0.05) and 12.46 (*p* ≤ 0.01) fold increase respectively when compared to normal stroma. Analysis of stroma enriched samples also revealed that SPARC and COLIA1 expression in the stromal compartments of both primary and metastatic tumour were highly significant (*p* ≤ 0.001) while these targets were only significantly enriched in metastatic tumours in whole biopsy analysis.

### Immunohistochemical validation of RNA-Seq results

Due to the rarity of Krukenberg tumours, we used archival samples of Krukenberg tumors for our immunohistochemical validation. Utilizing IGFBP7 as a representative marker, we stained and analyzed the epithelial and stromal components of each tissue microarray (TMA) core. Representative staining intensities are shown in Fig. 5c. All 14 stromal samples revealed high IGFBP7 staining while 11 out of 14 epithelial samples exhibited high staining (*p* = 0.0961) (Fig. 5d). Although our results did not reach significance possibly due to the small sample size, we confirmed the spatial expression of IGFBP7 thus validating the accuracy of LCM.

## Discussion

Whole tumour biopsies are typically used in conventional techniques of RNA-Seq, limiting the possibility of further differentiation of specific gene expression in various tumour cell types. Furthermore, conventional methods typically require the capture of a significant number of cells or numerous cycles of RNA amplification to generate ample quantities of RNA for downstream experiments, which may inadvertently introduce artifacts especially when initial RNA quantities are small^26–28^. In order to precisely characterize genomic aberrations in a tumour population, it is integral to acquire pure genetic material from cancerous cells with no or minimal contamination of neighbouring cells. LCM is a powerful high throughput technique which allows for the isolation of pure cell populations from fresh frozen or paraffin-embedded tissue sections and dissociated cultured cells^29^. It allows for the microscopic isolation of distinct cell types from tissue sections that may subsequently be utilized in gene expression analyses^30^. Additionally, LCM enables the isolation of homogeneous populations of cells from histologically complex tissues sections^31^. This approach results in satisfactory quality and quantity of RNA for subsequent experiments, such as microarray analyses^32,33^. It is a valuable technique that permits extraction of deeply embedded cells from tissues and has multiple advantages compared to conventional techniques.

Likewise, in this report, we have described and validated a workflow that combines LCM with RNA-Seq for a robust and efficient approach to RNA sequencing for NGS. Through our optimization, we were able to obtain high-quality RNA from LCM-derived matched tumour samples validated by RNA quality assessment platform, qPCR and IHC. The methodology of sequencing minute amounts of RNA obtained from LCM and RNA-Seq data generated from in-depth sequencing of microdissected tumour specimens was able to demonstrate high-quality RNA with minimal degradation as reflected by high RIN values in conjunction with acceptable quality control performance. Furthermore, qPCR and IHC data highlighted the difference in the expression profile of selected targets between whole tumour biopsies and the same samples subjected to tissue microdissection.

We have successfully demonstrated that in-depth analysis of tumour compartments and normalisation using matched tissue samples from CRS allows for the potential of identification of putative therapeutically relevant biomarkers that would otherwise not have been identified from conventional genomic sequencing approaches. The novelty of this paper lies in the analysis of multiple sampling of normal mucosal, stromal and epithelial components in the same patient to correct for innate biological differences. Notably, there is currently no literature on the use of LCM to reproducibly isolate high-quality RNA from systematic collection of matched tumours and its downstream applications specifically in colorectal and Krukenberg tumours. Furthermore, there is a lack of publicly available data, such as on TCGA, which are matched. As such, differences in gene expression in unmatched normal and tumour samples may not be truly representative of upregulated genes or signaling pathways in malignant cells. For example, the differing results of SPARC and COLIA1 expression in the microdissected vs non-microdissected samples highlight the utility of LCM for accurate target calling in molecular studies. Using conventional whole biopsy sequencing, SPARC and COLIA1 were identified as metastatic markers, but were not significant in the primary tumour. Harnessing the strength of LCM, the enriched samples showed that both SPARC and COLIA1 are significantly enriched in both primary and metastatic tumours, highlighting that these markers are biological relevant in both settings. Whole tumour biopsies may not provide enough resolution to identify important targets that might be hidden in sub-compartments of tissues. Using whole tumour sequencing may lead to the overlook of common profiles in primary and metastatic tumours that reveal conserved signaling pathways. These observations suggest that key molecular markers may be masked by disparate gene expression levels in different tissue compartments, highlighting the importance of tissue composition in the generation of molecular data. LCM allows for specific components to be dissected and subsequently a more accurate gene expression profile in separate components of the tumour sample^34,35^. This technique potentially provides greater insights into early genetic changes found within distinct tissue compartments enabling the investigation of molecular events that underlie the progression of malignant lesions^36^. Hence, LCM-based analyses of patient-derived clinical samples may be a routine and useful method for the identification of underlying pathological causes of malignancies for diagnostic and therapeutic applications.

We would like to emphasize that this manuscript serves as a methods paper of utilizing LCM to uncover targets that would be missed with conventional bulk sequencing, with qPCR and IHC data for technical validation. We do not claim that we have identified key differentially expressed genes in peritoneal metastasis in general. The current study is statistically underpowered for the identification of robust differentially expressed genes that dictate the biology of peritoneal metastases, and doing so would in turn generate multiple false positive results. To identify key differentially expressed genes that define the transcriptomic landscape of peritoneal metastasis will require a different study design and a larger sample size based upon careful statistical power calculations^37^, a possibility by consortiums such as TCGA.

Nonetheless, there are limitations to our approach. Even if RNA-Seq is able to pick up differentially expressed targets, validation using qPCR still requires selection of targeted genes. As such, several key differentially expressed genes may not be detected and evaluated. Furthermore, availability of LCM equipment and downstream molecular applications are less efficient with formalin-fixed paraffin-embedded (FFPE) tissue samples compared to ethanol fixed and live cultured cells samples^38,39^. RNA-Seq is mainly limited to snap frozen samples due to inherent problems with FFPE tissues where the fixation and embedding processes directly modify and degrade RNA^40^. Moreover, freshly acquired specimens are technically challenging due to size constrains^41^. As the amount of RNA isolated with LCM is minute, sample degradation needs to be kept to a minimum^10^. However, degradation may also occur during staining procedures which are critical for the identification of cells for capture. Studies have reported a substantial drop in RNA levels in LCM samples from tissues following exposure to staining solutions during immunocytochemistry^42^, although there was no difference in RNA yield when immunohistostaining was carried out in the presence of RNase inhibitors in all solutions except wash buffers^43^. Hence, given limited tissue quantity of early neoplastic lesions, maximising genetic and molecular information obtained from these tissue specimens represents a vital technical challenge.

## Conclusion

These findings suggest that in the era of NGS and large consortiums, a fraction of samples should be subjected to LCM before transcriptomics analysis to enable the identification of robust targets. We present here, in principle, a method that reliably produces high quality RNA from fresh-frozen colorectal and Krukenberg microdissected matched specimens that are amenable to transcriptome profiling that has not been demonstrated before. We were able to obtain high-quality RNA from LCM-derived tumour samples validated by RNA quality assessment indicators and qPCR amongst paired samples of primary, metastatic and normal tissue. We further showed that RNA-Seq from the combination of matched trio (normal-primary-metastatic) samples allows for the potential identification of putative therapeutically relevant biomarkers that can provide insights into tumor evolution and improve data integrity as compared to public databases of TCGA which only describes the macroscopic findings of cancers. Hence, this study demonstrates the usefulness of LCM for transcriptome analysis that enables comparisons of discrete cell populations residing within malignant tissues procured from intact tissue sections, a methodology that could be harnessed for biomarker discovery. Moving forward, this workflow may be practiced on other tissue types and will enhance research on neoplastic progression, epithelial-stromal interactions and unique cancer biomarkers.

## Methods

### LCM and RNA extraction

Four patients with Krukenberg tumours of colorectal origin were identified and recruited for this study. Multiple samples of matched normal mucosa, primary colorectal tumours and ovarian metastasis (Krukenberg tumours) as described in Table 1 were systematically harvested for this study. In total, 20 samples of colorectal and Krukenberg tumours with paired adjacent normal colonic mucosa were subjected to LCM and subsequently used for gene expression analysis. This study was approved by the SingHealth Centralized Institutional Review Board (CIRB Ref: 2015/2479/F) and informed consent was obtained from patients. All the experiments were performed in accordance with the relevant guidelines and regulations. Tissue sections of 10μm were cut and mounted onto plain glass slides for manual microdissection or PEN membrane slides (Life Technologies, Carlsbad, CA, USA) for UV LCM. The frozen sections were briefly washed in nuclease-free water and stained with 3:1 cresyl violet acetate: eosin stain (Sigma Aldrich; Missouri, USA; C5042 and HT110132) before undergoing dehydration through graded alcohols and xylene. Thorough dehydration helps reduce the activity of endogenous RNase enzymes. To minimise RNA degradation, duration of tissue handling remained short. Using the H&E image with pathological annotations (Supplementary Fig. S1) as a reference, cutting outlines were drawn meticulously around specific cells to prevent tissue contamination. Consecutive sections were used for LCM and photo documentation. Laser-captured cells were collected on LCM Macro CapSure caps (Life Technologies, Carlsbad, CA, USA) using the Arcturus XT LCM instrument (Life Technologies, Carlsbad, CA, USA).

Total RNA was extracted from all 20 microdissected tissues and 3 whole biopsies which were not subjected to LCM using the QIAgen RNAeasy Micro Kit (Qiagen, CA, USA), according to the manufacturer’s instructions. All 9 samples were stored in nuclease-free tubes and stored at −80 °C. To reduce sample degradation, the bench top and instruments required for all laboratory procedures such as sample preparation, microdissection, and RNA extraction were cleaned using RNase AWAY® (Life Technologies, Carlsbad, CA, USA) to minimise atmospheric RNase enzyme.

### RNA quality assessment

Aliquots of each RNA pool were analysed for quality and quantity on a Bioanalyzer RNA Pico chip (Agilent). The quality and quantity of isolated total RNA were evaluated using the 2100 BioAnalyzer (Agilent Technologies, Palo Alto, CA) with RNA 6000 Pico LabChip kit (Agilent Technologies, Santa Clara, CA). 1 µl of each isolated RNA sample was analysed with RNA 6000 Pico Assay employing RNA Pico LabChips (Agilent Technologies). Briefly, the quality of each RNA sample was approximated by the fragment size distribution, including two peaks correlating to 18 S and 28 S ribosomal RNAs, and a signal in the small molecular weight area, corresponding to small RNAs. This distribution was correlated to an extensive database of RNA samples and a RIN was tabulated. The resultant electropherograms were used to determine RNA integrity and concentration. RIN was calculated by the instrument where a value of 10 denotes intact RNA and a value of 1 indicates complete degradation.

### RNA library preparation and sequencing

Nugen Ovation RNA-Seq System (Nugen, CA, USA) was used to prepare the sequencing library. The amount of RNA used ranged from 3–60 ng. As defined in the user’s manual, amplification of both mRNA and non-polyadenylated transcripts makes the Ovation FFPE RNA-Seq Multiplex System (Nugen, San Carlos, CA, USA) ideal for amplification before the use of next generation sequencing (NGS). The libraries were checked using Agilent DNA1000 chip to confirm that all obtained profiles conform to what was expected. Six uniquely indexed libraries were pooled and loaded into each lane of flow cells. Sequencing was carried out via Illumina HiSeq 2500 (Illumina, San Diego, CA, USA) at 150 base pairs, paired end, and 50 million paired reads per sample as per the manufacturer’s protocol.

### FastQC validation

For quality assessment of raw sequencing reads, FASTQ files were analysed using the FASTQC tool using default settings^44^. To assess the quality of read alignments, the sequencing reads were first aligned to the human reference transcriptome (hs37d5) using the STAR aligner using default settings^45^. The BAM files were then processed using the RSeQC tool to produce various RNA-Seq diagnostic plots^46,47^. Briefly, transcripts were grouped quartiles based on their expression level. For each quartile, the degree to which transcript expression levels differed when using a random subset of reads as compared to when using the full set of reads was determined. This resampling process was repeated using varying fractions of the total reads. If sequencing depth is adequate, the relative error should be low and stable even when a subset of reads are used, indicating that saturation has been achieved. In this case, transcripts below the 25^th^ percentile of expression (“Q1”) display saturation, suggesting that even low-abundance transcripts have been accurately quantified. To assess the contamination rate of human ribosomal RNA (rRNA) in the libraries, sequencing reads were aligned to a reference comprising the sequences of known human ribosomal RNAs using BWA under default settings, and the extent of rRNA contamination inferred from fraction of reads in the library aligning to the rRNA reference^48^.

### RNA expression analysis and qPCR verification of selected gene targets

Using DNA nexus applications, reads were mapped using TopHat2 v2.0.12 and CuffDiff v2.1.1 on the genome version ucsc_hg19. Fragments per Kilobase of transcript per Million (FPKM) and fragment counts for each transcript, primary transcript and gene in each sample was normalized as part of CuffDiff. Data was further analysed in R (v 3.4.2). FPKM values from CuffDiff were used for principal component analysis (PCA), and a distance matrix (representing all the pair wise distances between all samples analysed) was visualised using the hopach library. As this was an exploratory analysis, targets selected for qPCR validation was based on biological significance. To verify the expression data derived from the RNA-Seq, we applied qPCR to the RNA extracted from microdissected tissues as well as matched biopsies which were not subjected to LCM in order to evaluate the quality of RNA-Seq analysis. We selected genes from the RNA-Seq data to represent increased expression unique to certain components or combinations of components (Supplementary Table S2) in order to validate the differences between whole samples and LCM samples. cDNA was synthesized using 100 pg of RNA with qSCript cDNA SuperMix (Quanta Biosciences, Beverly, MA) and processed on the SimpliAMP thermal cycler (Applied Biosystems, Thermo Scientific, Waltham, Massachusetts). PCR primers were first designed with Primer3 v0.4.0 and inputted into NCBI BLAST to check for specificity of the primers generated (Supplementary Table S3). *β*-Actin was used as a reference gene. qPCR was performed on the CFX-96 system (Bio-Rad Laboratories, Berkeley, California) in triplicates per sample using the iTaq universal SYBR Green supermix (Bio-Rad Laboratories, Berkeley, California). The data was normalized to *β*-Actin and analysed using one-way ANOVA.

### Immunohistochemistry (IHC)

A tissue microarray (TMA) was constructed using Krukenberg tumours collected from patients who were treated in the National Cancer Centre Singapore or Singapore General Hospital between 2004–2017 (n = 14). The constructed TMA block was sectioned into 5 µm sections and mounted onto slides. IHC staining of the TMA sections was performed using the BOND-MAX autostainer (Leica Microsystems, Wetzler, Germany) as per the manufacturer’s instructions. Briefly, sections were deparaffinized and rehydrated. Antigen retrieval was conducted at 100 °C, following which the slides were cooled down to room temperature and washed. In order to block endogenous peroxidase activity, sections were incubated in 3–4% (v/v) H_2_O_2_ for 15 minutes. To prevent non-specific binding, sections were blocked in 10% goat serum. They were then incubated with anti-IGFBP7 (ab171085, Abcam, Cambridge, UK) antibody for 20 minutes, following which the slides were rinsed and exposed to polymer for 5 minutes. Slides were then washed, treated with DAB-Chromogen detection reagent for 7 minutes and then rinsed again. Sections were subsequently exposed to haematoxylin for 5 minutes to counterstain the nuclei. Finally, slides were rinsed, dehydrated and mounted. Colon samples were used as positive controls while the negative control was derived by replacing the primary antibody with Bond^TM^ Antibody diluent.

### Evaluation of IHC staining

All stained sections were assessed by two independent scorers (WHN and JH) who were blinded to the outcome. Scoring for the epithelial and stromal components was conducted by stratifying the intensity of the stain to a value of 0 (no staining) to 3 (strong staining) if at least 10% of the target cells were positively-stained (Fig. 5c). In situations where there were discrepancies, a third researcher (HJL) assessed the sections independently to obtain the final score. t-test analysis was used to compare between the epithelial and stromal expressions.

## Supplementary information

Supplementary Information

## Data Availability

RNA sequencing raw data were deposited into the Gene Expression Omnibus (GEO) under the accession code GSE119968.
