# Supplementary material for: An Optimised Protocol Harnessing Laser Capture Microdissection for Transcriptomic Analysis on Matched Primary and Metastatic Colorectal Tumours
Source: Sci Rep. 2020 Jan 20;10:682. doi: 10.1038/s41598-019-55146-2 (PMC6971024; doi:10.1038/s41598-019-55146-2)
Supplement: Supplementary file 1 — Supplementary Information [file 41598_2019_55146_MOESM1_ESM.pdf]

AN OPTIMISED PROTOCOL HARNESSING LASER CAPTURE MICRODISSECTION ON MATCHED PRIMARY AND METASTATIC COLORECTAL TUMOURS

CHIN-ANN J. ONG[1*], QIU XUAN TAN[1*], HUI JUN LIM[1*], NICHOLAS B. SHANNON[1], WENG KHONG LIM[2], JOSEPHINE HENDRIKSON[1], WAI HAR NG[1], JOEY W.S. TAN[1], KELVIN K.N. KOH[1], SEETTHA D. WASUDEVAN[1], CEDRIC C.Y. NG[3], VIKNESWARI RAJASEGARAN[3], TONY KIAT HON LIM[4], CHOON KIAT ONG[3], OI LIAN KON[3], BIN TEAN TEH[3], GRACE H.C. TAN[1], CLARAMAE SHULYN CHIA[1], KHEE CHEE SOO[1], MELISSA C.C. TEO[1]**

[1] Division of Surgical Oncology, National Cancer Centre Singapore, 11 Hospital Drive, S169610

[2] Cancer and Stem Cell Biology, Duke-NUS Medical School, 8 College Road, Singapore 169857

[3] Division of Medical Sciences, National Cancer Centre Singapore, 11 Hospital Drive, S169610

[4] Department of Anatomical Pathology, Singapore General Hospital, 20 College Road, S169856

[*] **Equal contributions**

[**] **Correspondence author**

The authors declare no conflict of interest.

Correspondence to:

Adj Professor Melissa Teo Ching Ching

Division of Surgical Oncology

National Cancer Centre Singapore

11 Hospital Drive Singapore 169610

melissa.teo.c.c@singhealth.com.sg

+65 64368498

Supplementary Information

Supplementary Table S1: List of microdissected samples used in RNA-Seq analysis						
	**Normal colonic mucosa**		**Primary colorectal tumour**		**Krukenberg tumour**	
	**Stroma**	**Normal Epithelium**	**Stroma**	**Normal Epithelium**	**Stroma**	**Normal Epithelium**
Patient 1	1	1	1	1	1	1
Patient 2	1	1	1	1	2	2
Patient 3	0	0	1	1	1	1
Patient 4	0	0	0	0	1	1

**Supplementary Table S2:** Gene expression patterns of selected targets	
**Gene**	**Expression pattern**
SPINK1	Higher expression in tumour epithelium compared to normal epithelium
ERBB2	Higher expression in metastatic tumour epithelium compared to normal epithelium and primary tumour epithelium
COLIA1	Higher expression in tumour-associated stroma compared to normal stroma
SPARC, TIMP1, IGFBP7	Higher expression in metastatic stroma compared to normal stroma and primary tumour-associated stroma
ENO1	Higher expression in metastatic epithelium and metastatic stroma compared to the respective tissue compartments in normal mucosa and primary tumour
VEGFA, S100A11	Higher expression in tumour epithelium and stroma compared to respective tissue compartments in normal mucosa
PLA2G2A	Lower expression in tumour epithelium compared to normal epithelium
CEACAM1	Lower expression in tumour compared to normal mucosa

**Supplementary Table S3:** List of primers	
**Gene ID**	**Primer Sequence 5' to 3'**
GAPDH F	CAACAGCCTCAAGATCATCAG
GAPDH R	ATGGACTGTGGTCATGAGTC
β ACTIN F	TGTTTGAGACCTTCAACACC
β ACTIN R	AGGTAGTCAGTCAGGTCCCGGCC
ErbB2 F	ACTGGCCCTCATCCACCATA
ErbB2 R	GGTTGGCAGTGTGGAGCAG
SPINK1 F	TGTCTGTGGGACTGATGGAA
SPINK1 R	AGGCCCAGATTTTTGAATGA
CEACAM1 F	AAGCCCCAAATCAAAGCCAG
CEACAM1 R	CAGCATCCTCCCTCTTGACA
SPARC F	AAACTTTTGGGAGCACGGAC
SPARC R	ACCGATTCACCAACTCCACT
TIMP-1 F	CCTTCTGCAATTCCGACCTC
TIMP-1 R	GTATCCGCAGACACTCTCCA
IGFBP7 F	TGGGTGCTGGTATCTCCTCT
IGFBP7 R	TATAGCTCGGCACCTTCACC
COLIA1 F	ATGTGCCACTCTGACTGGAA
COLIA1 R	CTTGTCCTTGGGGTTCTTGC
ENO1 F	AAAGCTGGTGCCGTTGAGAAG
ENO1 R	AGCATGAGAACCGCCATTGAT
S100A11 F	TCTCCAGCCCTACAGAGACT
S100A11 R	TTCATCATGCGGTCAAGGAC

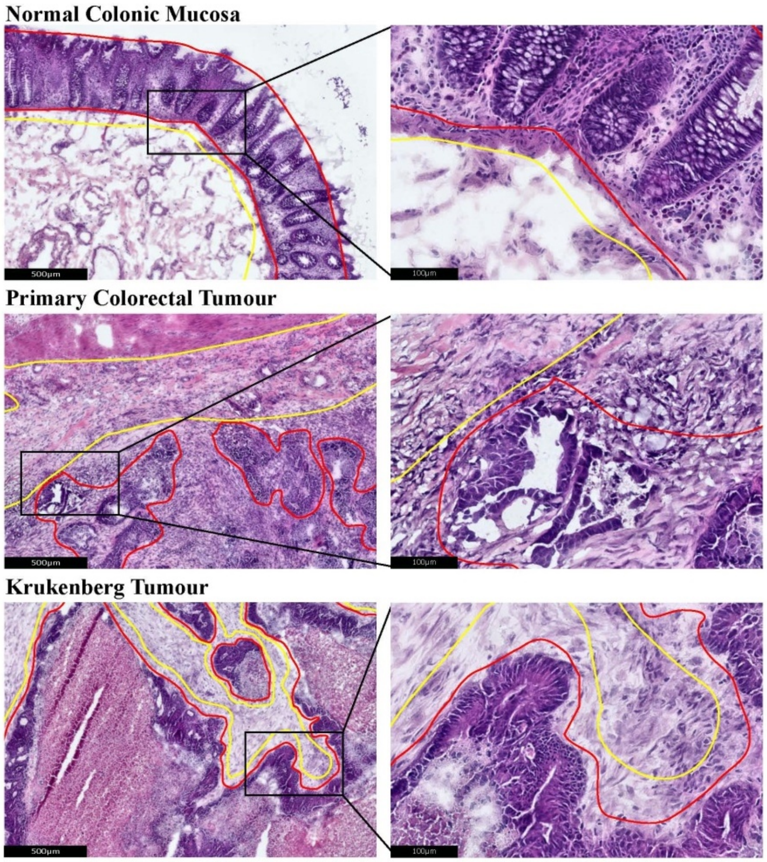

Supplementary Figure S1: H&E stained slides with pathology annotation of stroma and tumour regions for LCM.

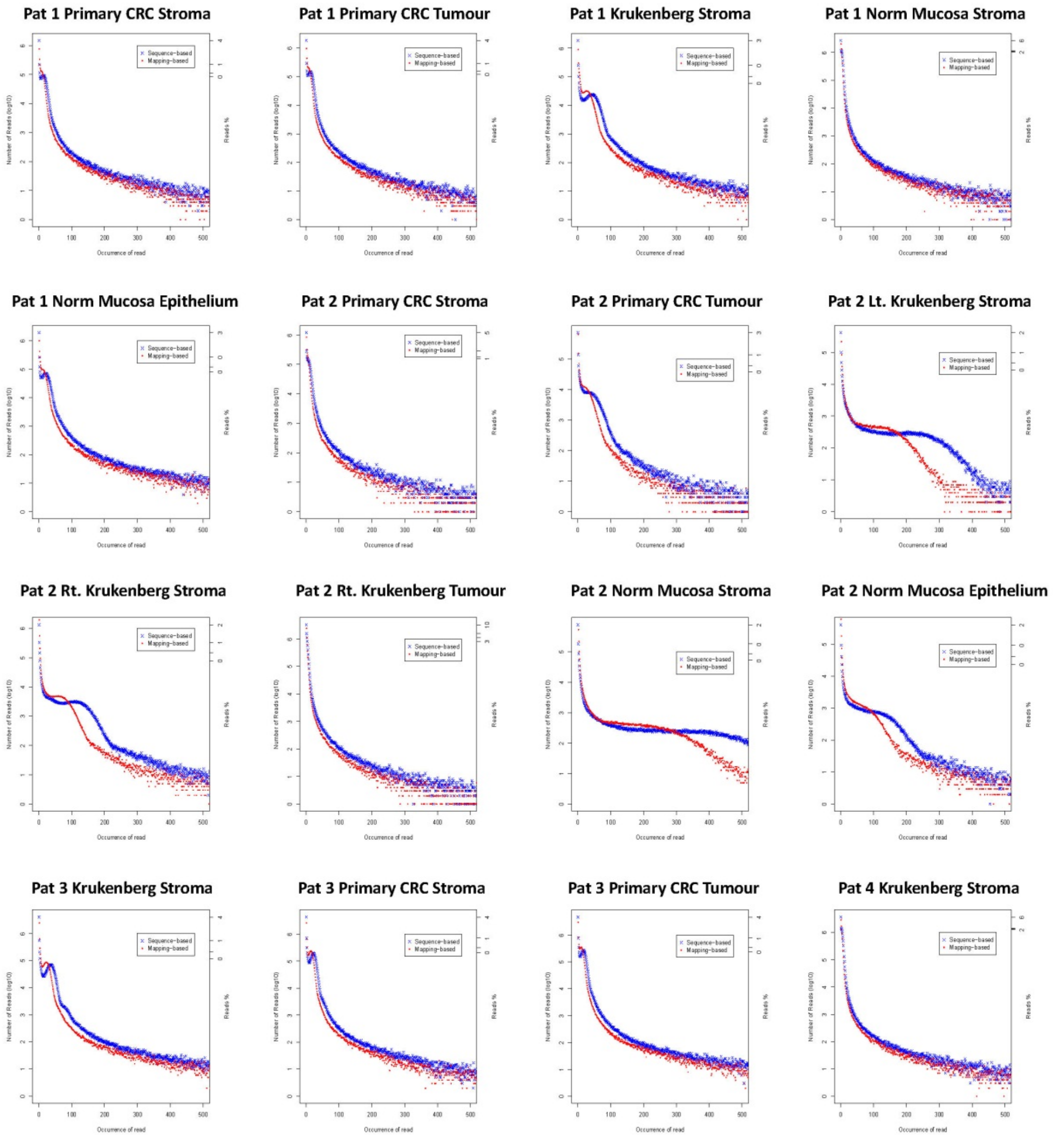

Supplementary Figure S2: (a)The distribution of duplicated reads relative to the total number of sequences for all libraries. The duplication levels relative to the total number of sequences show a distribution that is skewed to the right of the graph. This is expected of deeply sequenced enriched libraries with good library diversity, with the exception of a few samples which may have possible residual ribosomal RNA contamination as observed from the report of overrepresented sequences.

Pat 1 Primary CRC Stroma

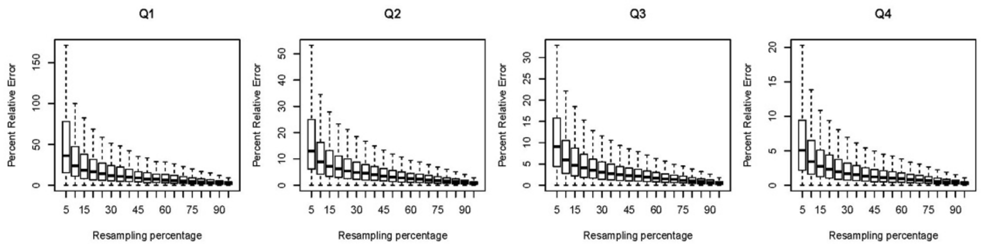

Pat 1 Krukenberg Stroma

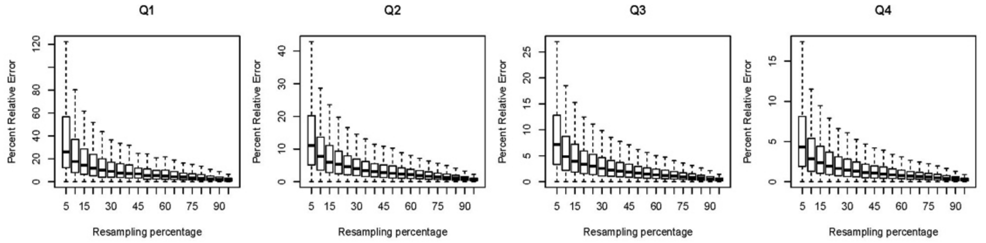

Pat 1 Krukenberg Tumour

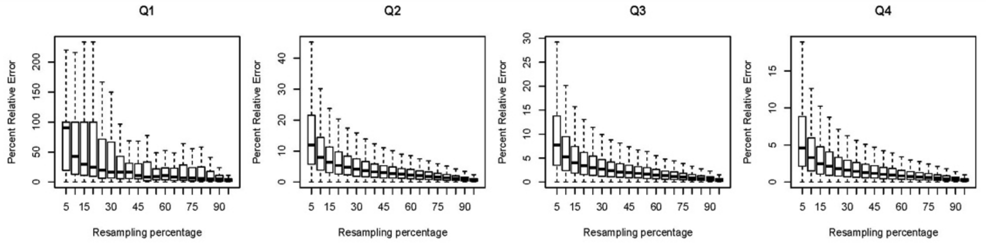

Pat 1 Norm Mucosa Epithelium

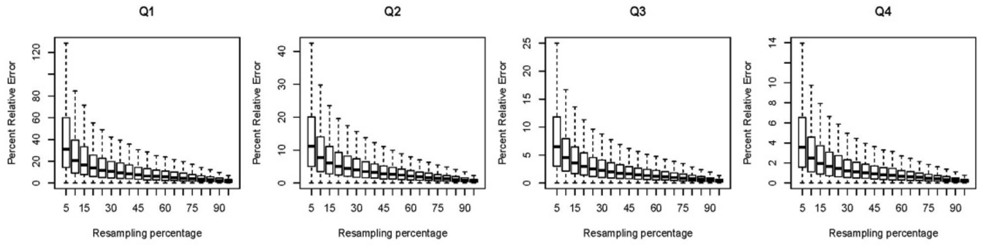

Pat 1 Norm Mucosa Stroma

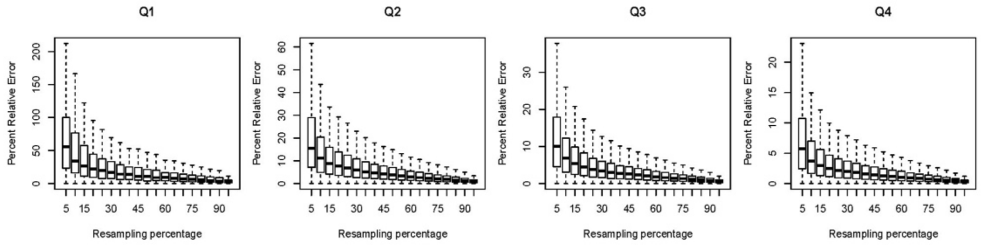

Pat 2 Primary CRC Stroma

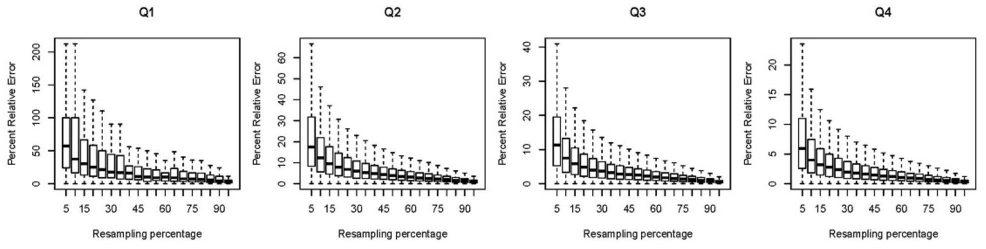

Pat 2 Primary CRC Tumour

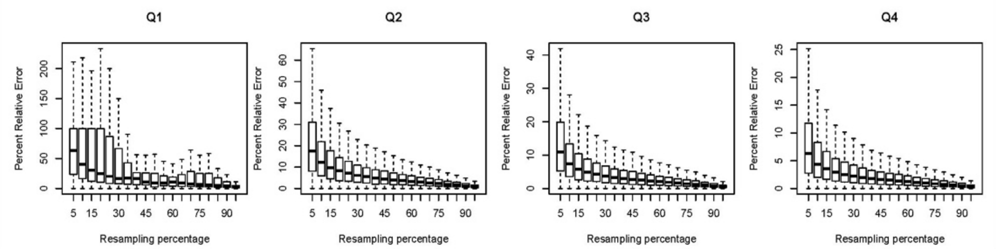

Pat 2 Lt. Krukenberg Stroma

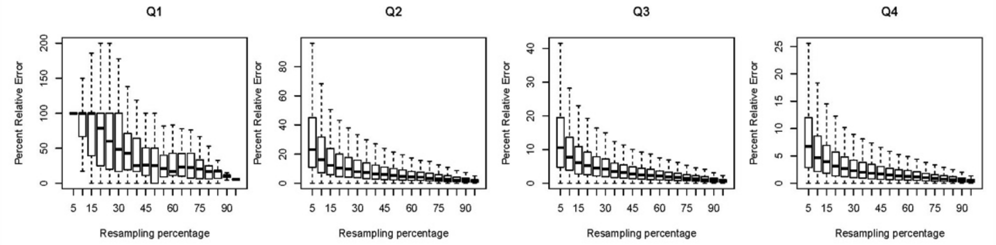

Pat 2 Lt. Krukenberg Tumour

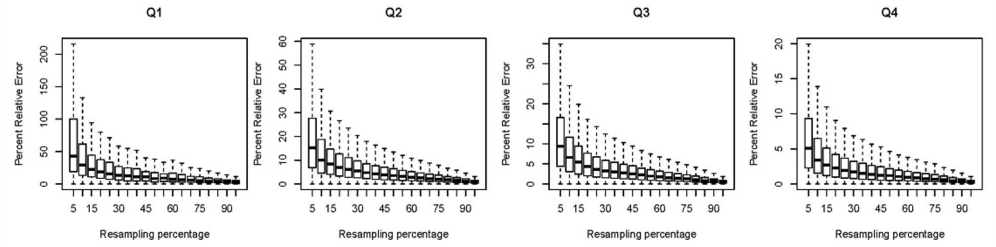

Pat 2 Rt. Krukenberg Stroma

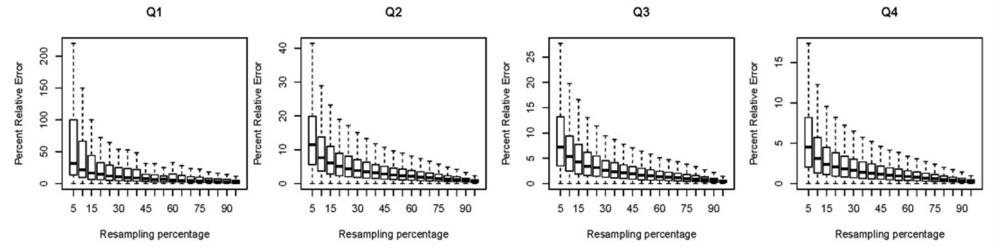

Pat 2 Rt. Krukenberg Tumour

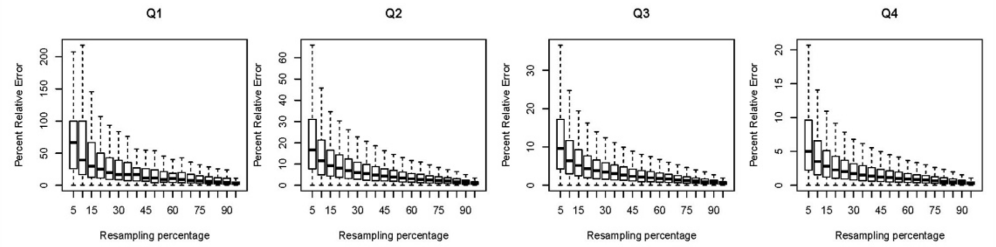

Pat 2 Norm Mucosa Epithelium

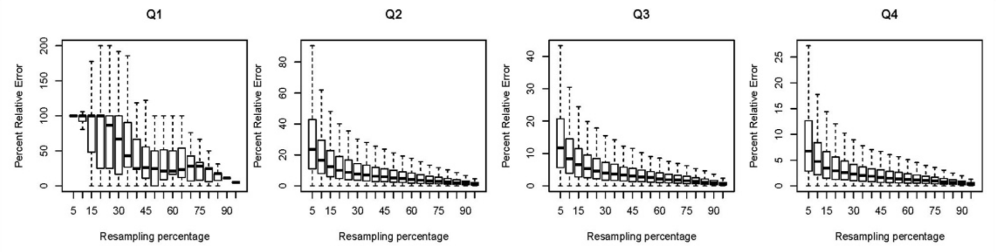

Pat 2 Norm Mucosa Stroma

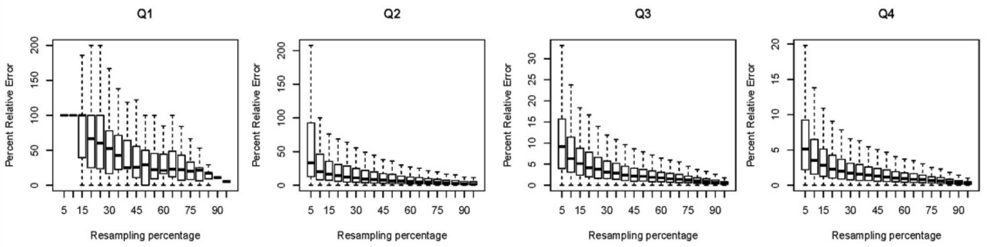

Pat 3 Lt. Krukenberg Stroma

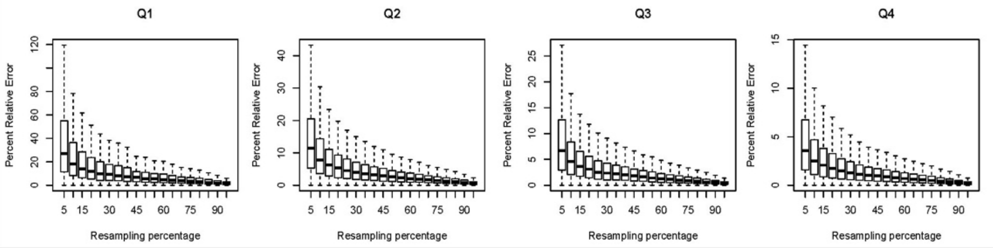

Pat 3 Lt. Krukenberg Tumour

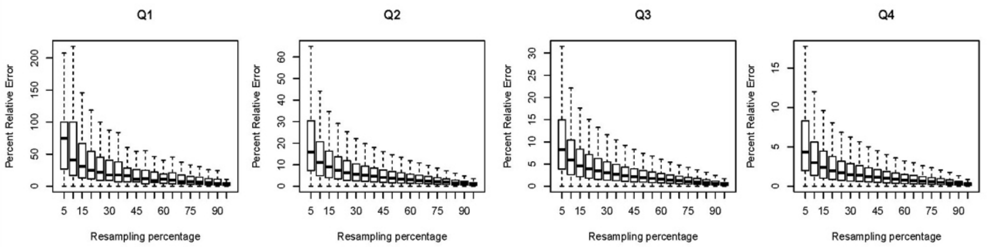

Pat 3 Primary CRC Stroma

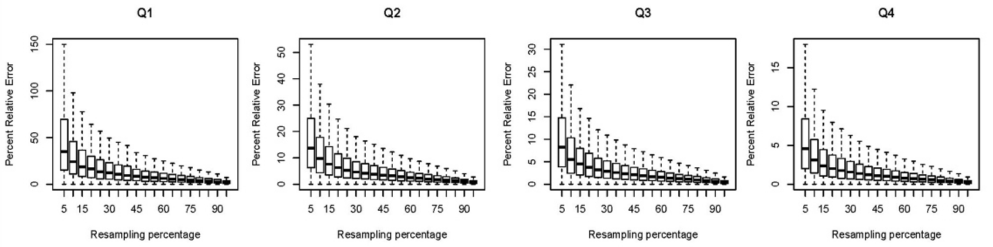

Pat 3 Primary CRC Tumour

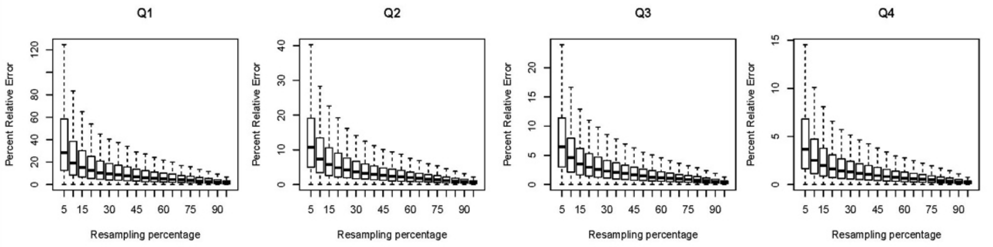

Pat 4 Krukenberg Stroma

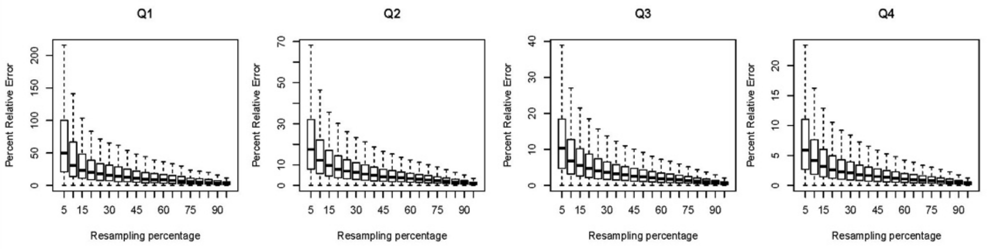

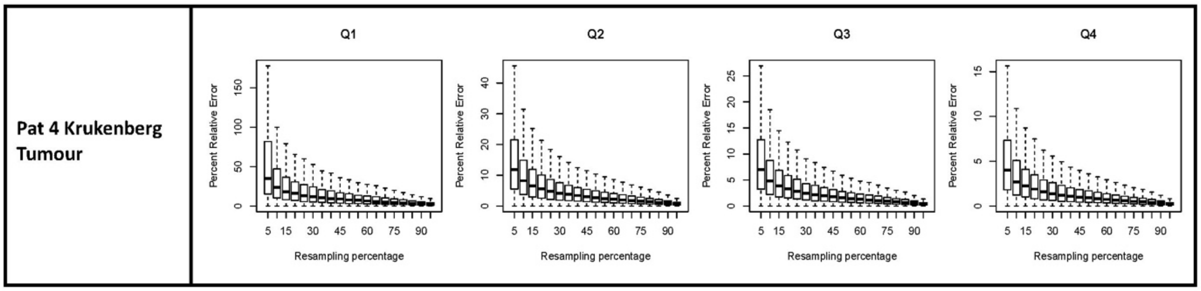

Supplementary Figure S2: (b) Saturation plots of the respective samples illustrate sufficient sequencing depth across all samples.

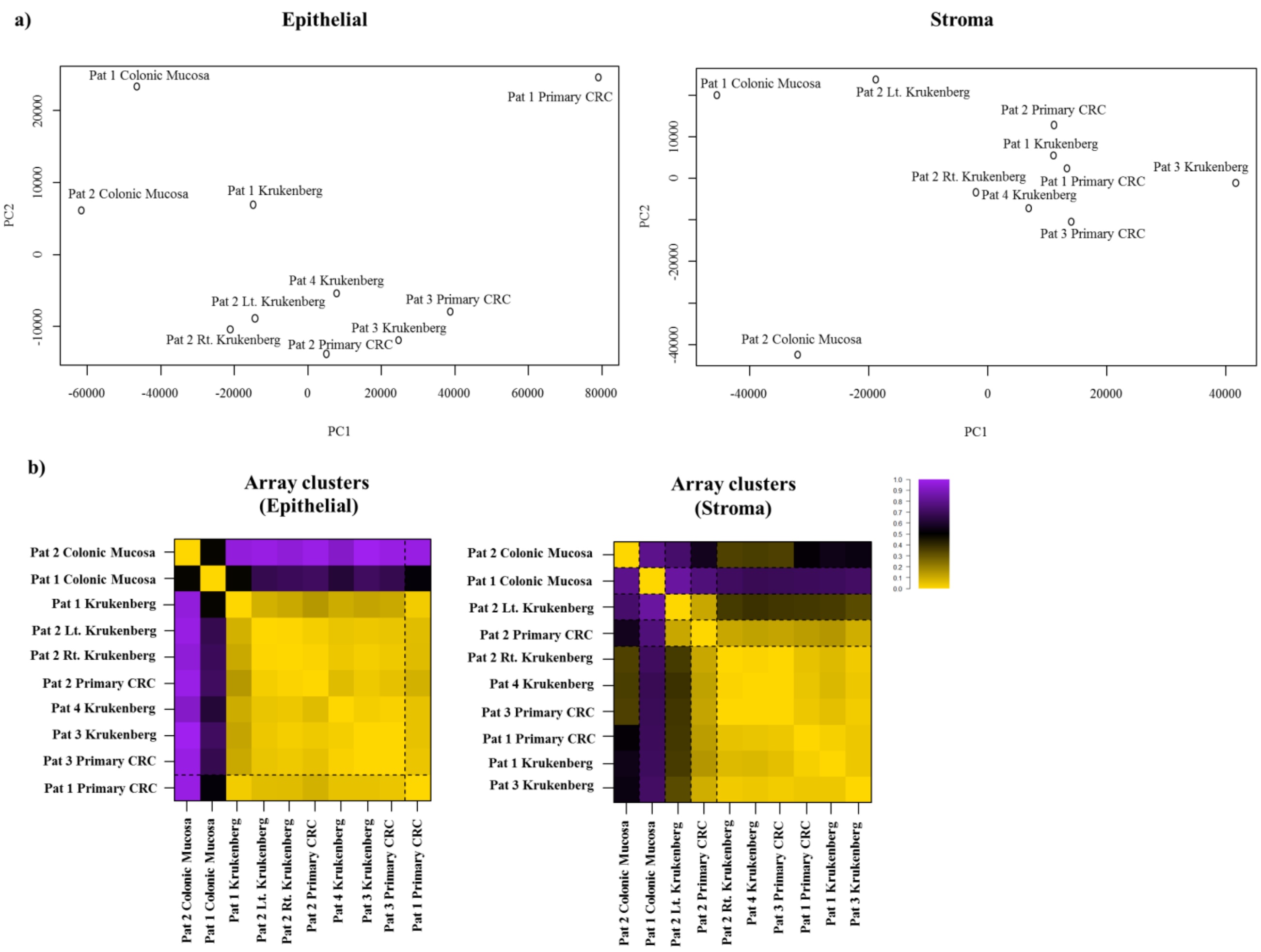

Supplementary Figure S3: (a) Principal component analysis (PCA) of the epithelium and stroma derived from all the microdissected samples. (b) Distance matrix of epithelium and stroma of all the microdissected samples. *Purple: Different, Yellow: Identical*

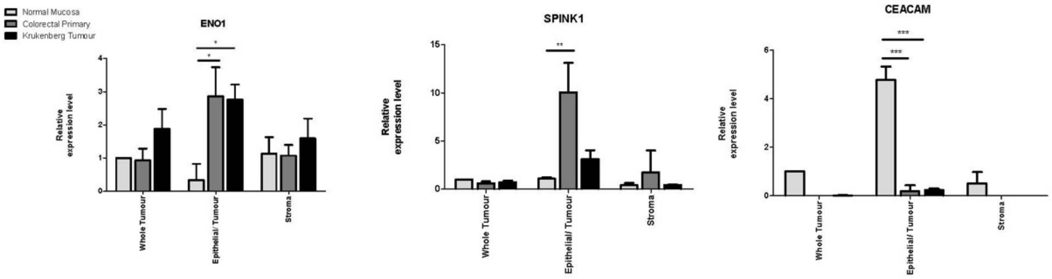

Supplementary Figure S4: Validation of RNA-Seq data by qPCR analysis of LCM samples from patient 1. Results illustrating the expression patterns for selected targets. Results are the mean of three biological replicates and standard deviations are shown.
